# Hair regrowth and maintenance in alopecia universalis patient treated with nonablative Er:YAG laser

**DOI:** 10.1002/ccr3.4948

**Published:** 2021-11-12

**Authors:** Madison McCarthy, Doris Day, Iva Talaber

**Affiliations:** ^1^ Doris Day M.D New York USA; ^2^ Fotona d.o.o. Ljubljana Slovenia

**Keywords:** alopecia areata, alopecia universalis, hair regrowth, laser treatment

## Abstract

Hair regrowth with no adverse effects following nonablative 2940‐nm Er:YAG laser treatment in alopecia universalis patient resulted in high patient satisfaction and compliance. As the main challenge in alopecia universalis is maintenance of regrown hair, patient compliance associated with this treatment might represent an advantage over traditional treatment modalities.

## Graphical abstract

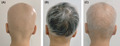

## 1 INTRODUCTION

Alopecia areata (AA) is a common, inflammatory, nonscarring type of hair loss that presents as well‐demarcated, round patches of hair loss, sometimes progressing to include all scalp or body hairs.^1^ AA can be classified into three main clinical phenotypes, including patchy type AA (AAP), alopecia totalis (AT), and alopecia universalis (AU), based on the severity and areas of hair loss.^2^

Alopecia totalis (AT) occurs when scalp and face hair such as eyebrows, eyelashes, and beard are affected, while the term alopecia universalis (AU) refers to cases of entire body hair loss.^3^ AA affects people of all age groups, sexes, and ethnicities and represents a source of frustration due to the unpredictable nature of the disease for which there is currently no definitive treatment.^4^ The etiology of AA is not completely understood,^5^ but it supposedly results at least in part from a loss of immune privilege in the hair follicle, autoimmune‐mediated hair follicle destruction, and the upregulation of inflammatory pathways.^4^ Many patients do not find existing therapies helpful and seek mental health treatments to alleviate the psychological burden of this disease.^6^

Treatment options are aimed at hair regrowth and control of remissions^3^ and include injectable corticosteroids, systemic immune suppressants, psoralen plus ultraviolet light A radiation (PUVA), minoxidil, and topical irritants, such as anthralin and diphencyprone, aimed at blocking the T‐cell‐mediated hair loss process using immunosuppression or competitive immunostimulation.^7^ Effectiveness of immunotherapy may be proportional to the severity of hair loss, as was shown in the case of topical diphencyprone having the greatest effect in patients with less than 50% hair loss and smallest effect in patients with AT and AU.^8^ The 308 nm excimer laser has been reported to be effective for alopecia areata, with the phototherapeutic mode of action of cutaneous immunosuppression by UVB light.^9^ At the other end of the spectrum, infrared light has also been shown to promote hair regrowth in patients with patchy AA^10^ and AA recalcitrant to other treatment.^7^ Although the underlying mechanism is poorly understood, the anti‐inflammatory effect of infrared irradiation demonstrated in various in vivo and in vitro conditions might correct the peribulbar inflammation of alopecia areata.^7^ In case of ablative fractional 2940‐nm Er:YAG laser, it has been reported to induce hair growth in a murine model^11^ as well as in human patients with AA.^12^ In a recent study,^13^ a nonablative fractional 1540‐nm Er:YAG laser resulted in more hair regrowth compared to control patches at 6 and 12 weeks follow‐up.

In this study, a nonablative 2940‐nm Er:YAG treatment in SMOOTH™ mode was used to treat a female patient with AU. The SMOOTH™ mode denotes trains of short subablative pulses of laser light, which is absorbed in the most superficial (<10 µm) layer of the skin, leaving only heat to diffuse to deeper layers (400 µm). This treatment modality results in paracrine signaling that activates fibroblasts and initiates regenerative processes in the skin^14^, ^15^ and has already been used for stimulation of hair growth.^16^

## 2 CASE REPORT

**FIGURE 1:**
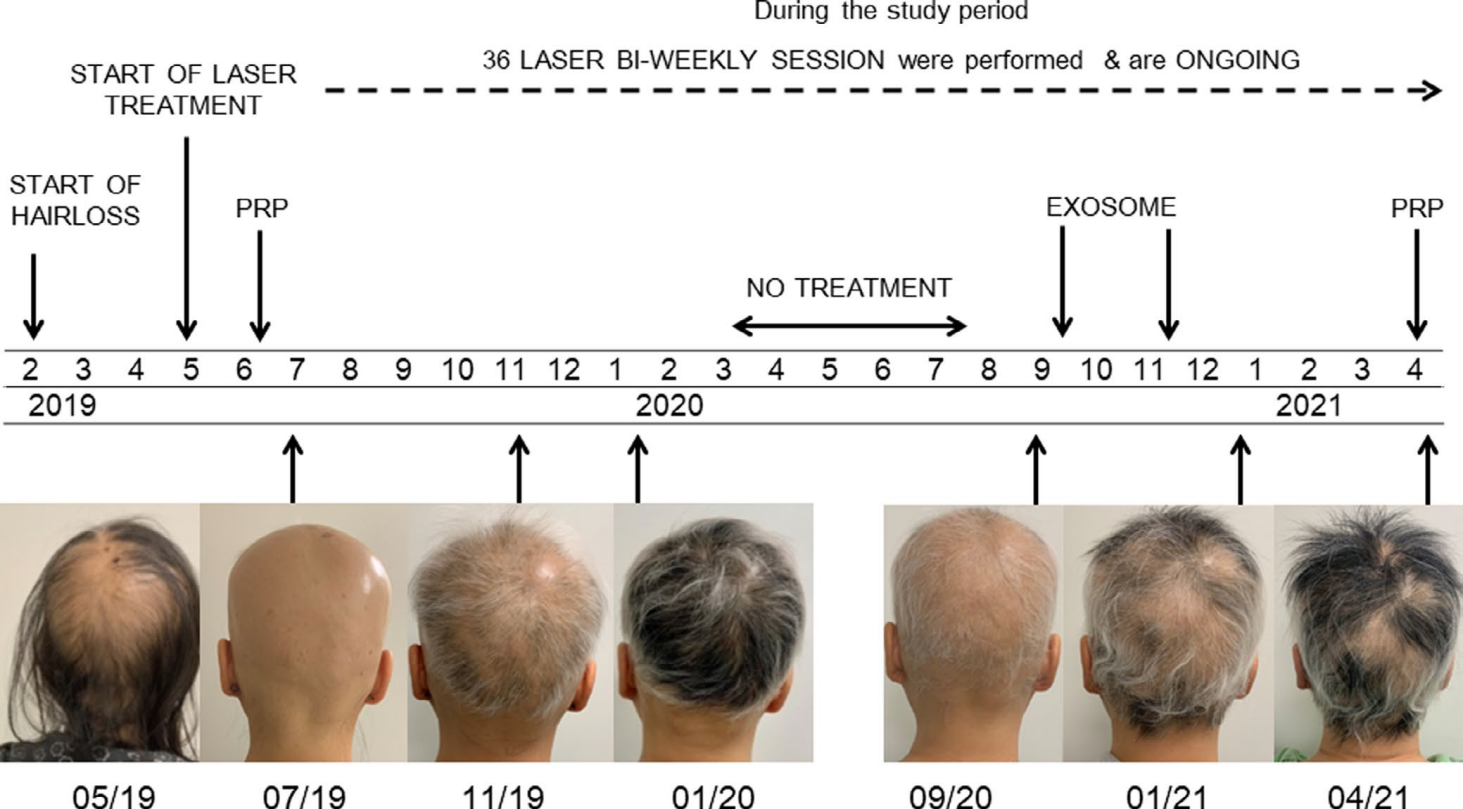
Timeline of study

A 52‐year‐old woman with a 2‐year history of AU presented to the dermatology clinic as her alopecia had progressed over the past months. At initial assessment, the patient's hair loss was assessed as Grade 3 by Ludwig classification (Figure 1). The patient first started experiencing hair loss in February 2019. At this time, she noticed overall thinning and hair shedding throughout the scalp. The patient began taking Allegra and Nutrafol Women in April 2019 and continued throughout the study period.

In the period May 2019–April 2021, the patient received 36 treatment sessions with the 2940 nm nonablative Er:YAG laser (SP Dynamis, Fotona, Slovenia) with SMOOTH TM mode using fixed parameters (7 mm spot size, 7.00 J/cm^2^ pulse fluence, 3.3 Hz frequency). The PS03 laser handpiece was moved in a cross‐hatched pattern across the scalp for 3 passes, and the total amount of energy delivered was between 450 and 600 J per session. The total area of hair loss was treated.

In addition to the laser treatment, the patient was treated with PRP (June 2019, April 2021) and exosomes (in September and November 2020). The PRP was obtained with an Eclipse PRP kit. The patient's blood was drawn using a 22 mL collection tube and centrifuged at 3200 RPM for 10 minutes. Extracted PRP was distributed into separate 3 mL syringes containing 0.15 cc of lidocaine each. Prepared PRP solution was injected in 0.1–0.2 cc aliquots subcutaneously into the patients scalp with a 30G needle, starting at the frontal hairline and moving posteriorly at 1 cm increments. The exosome treatments were performed using Exovex, which is a placental MSC stem cell‐derived product containing isolated and concentrated exosomes suspended in 0.9% saline. Exovex was injected to the entire affected scalp area, using 5 1 ml syringes.

Potential hair regrowth was documented photographically. No laser‐related adverse effects developed during or after the treatment. The patient assessed pain level as a 2 on a 10 point VAS scale and satisfaction as a 3 on a 3‐point scale.

Over the course of the treatment, there was a continual gradual increase in hair growth. After 18 biweekly laser sessions, the treatment was stopped due to COVID and lockdown restrictions for a period of five months. In this time, the patient experienced increased shedding. Upon continuation of laser treatment, hair growth increased again.

## 3 DISCUSSION

**FIGURE 2:**
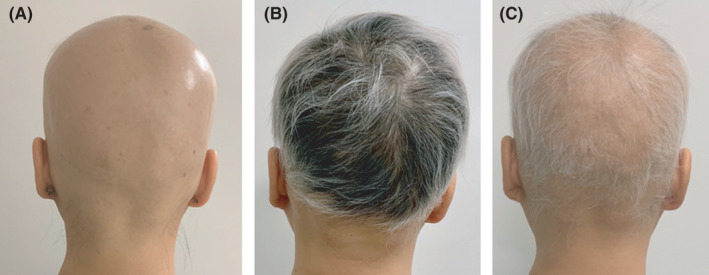
Representative photographs of the patient (A) during the most severe form of AU, (B) after 18 laser sessions, and (C) after 5 months break in laser treatment

As evident from the photographs taken at several time points during the 36 laser sessions (Figure 1), the patient experienced extensive hair regrowth. There was a lag between the start of the treatment and the initial hair growth, as hair shedding was still progressing after the first four laser sessions, during which PRP was also administered. Notable hair regrowth was first observed after the sixth session and gradually increased with consequent biweekly sessions. During the first 6 months, 18 sessions were performed and the alopecia condition dramatically receded from the most severe stage of alopecia (Figure 2A) to marked hair regrowth (Figure 2B). Notably, in this period, the patient was treated only with the laser therapy, demonstrating the effectiveness of repeated sessions of nonablative Er:YAG laser treatment in alopecia universalis.

The following biweekly laser sessions were interrupted for a period of five months (between March 2020 and July 2020) because of COVID and lockdown restrictions, during which increased hair shedding was observed resulting in decreased hair coverage (Figure 2C). Gradual increase in hair growth was observed again after the patient continued with laser treatments and received additional therapy with exosomes. The nonablative 2940‐nm Er:YAG treatment in SMOOTH™ mode seems to be effective for treatment of hair loss and maintenance of hair regrowth in alopecia universalis, possibly involved in prevention of condition progression. Although the effect of the adjunctive treatment modalities was not assessed, the contributing effect of PRP and exosome therapy cannot be excluded. No laser‐related side‐effects developed during or after the treatment. The procedure was not painful (2/10) for the patient, who rated her satisfaction with the highest score (3/3). Biweekly treatment is ongoing.

In contrast to androgenetic alopecia, where longer term preservation of the effect was shown for the nonablative 2940‐nm Er:YAG,^17^ maintenance sessions in alopecia universalis seem to be more important. This is in line with the conclusions of a study of the 1550‐nm nonablative fractional erbium–glass laser combined with topical minoxidil, where complete hair regrowth was observed after 10 sessions, but relapsed to baseline levels at 1 year follow‐up.^18^

The main challenge in AA/AT/AU treatment is maintenance of regrown hair.^3^ The repetitive nature of topical immunotherapy with its frequent side effects, including eczema, severe urticaria, dermographism, and sleep disturbances, results in low patient compliance. Consequently, recurrences of hair loss are common and unfortunately, more recalcitrant to treatment.^3^ Patient compliance might represent an important advantage of the nonablative 2940‐nm Er:YAG therapy over the traditional treatment modalities, as it demonstrates efficiency with an absence of adverse effect, and a high level of patient comfort and satisfaction. Additional studies with long term follow‐up are needed to further corroborate the efficacy, safety, and patient compliance on a larger number of patients. Furthermore, patients with AA/AT/AU could greatly benefit from studies aimed at assessing the optimal interval between laser sessions for the maintenance of regrown hair.

## CONFLICT OF INTEREST

The authors declare no conflict of interest.

## AUTHOR CONTRIBUTIONS

Author 1: Conceptualization of study, interpretation of clinical data, writing of the manuscript. Author 2: Conceptualization of study, interpretation of clinical data. Author 3: Interpretation of clinical data, writing of the manuscript, figure preparation.

## ETHICAL APPROVAL

All procedures performed in studies involving human participants were in accordance with the ethical standards of the 1964 Helsinki Declaration and its later amendments or comparable ethical standards.

## CONSENT

The patient signed an informed consent form after understanding the nature of the trial.

## Data Availability

The data that support the findings of this study are available on request from the corresponding author. The data are not publicly available due to privacy or ethical restrictions.
